# Molecular cloning and expression analysis of a zebrafish novel zinc finger protein gene *rnf141*

**DOI:** 10.1590/S1415-47572009005000062

**Published:** 2009-09-01

**Authors:** Wenqian Deng, Huaqin Sun, Yunqiang Liu, Dachang Tao, Sizhong Zhang, Yongxin Ma

**Affiliations:** Department of Medical Genetics, West China Hospital Division of Morbid Genomics, State Key Laboratory of Biotherapy, Sichuan University, Chengdu, SichuanPeople's Republic Of China

**Keywords:** *rnf141*, *zebrafish (Danio rerio)*, development, zinc finger protein

## Abstract

*ZNF230* is a novel zinc finger gene cloned by our laboratory. In order to understand the potential functions of this gene in vertebrate development, we cloned the zebrafish orthologue of human *ZNF230*, named *rnf141*. The cDNA fragment of *rnf141* was obtained by rapid amplification of cDNA ends (RACE). The open reading frame (ORF) encodes a polypeptide of 222 amino acids which shares 75.65% identity with the human *ZNF230*. RT-PCR analysis in zebrafish embryo and adult tissues revealed that *rnf141* transcripts are maternally derived and that *rnf141* mRNA has a broad distribution. Zygotic *rnf141* message is strongly localized in the central nervous system, as shown by whole-mount *in situ* hybridization. Knockdown and over expression of *rnf141* can induce abnormal phenotypes, including abnormal development of brain, as well as yolk sac and axis extendsion. Marker gene analysis showed that *rnf141* may play a role in normal dorsoventral patterning of zebrafish embryos, suggesting that *rnf141* may have a broad function during early development of vertebrates.

## Introduction

The zinc finger gene family, one of the largest gene families in mammals, is defined by a conserved cysteine and histidine rich domain essential for the binding of zinc ions (Freemont 1993; Klug and Schwabe, 1995). This gene family can be divided into several subfamilies, including ring finger, C2H2, glucocorticoid receptor, GATA1, GAL4, and LIM (Barlow *et al.*, 1994; Borden and Freemont, 1996; Hammarstrom *et al.*, 1996).

In accordance with their diverse structures, zinc finger proteins have been assigned multiple functions, including DNA recognition, transcriptional activation, RNA packaging, regulation of apoptosis, ubiquitination and many others (Coleman, 1992; Wolfe *et al.*, 2000; Laity *et al.*, 2001; Vazquez *et al.*, 2007). More than 20 different zinc finger genes located on sex chromosomes or autosomes have been proposed to play a regulatory role in mammalian spermatogenesis (Noce *et al.*, 1992; Pieler and Bellefroid, 1994; Yan *et al.*, 2002).

The human *ZNF230*, which maps to the short arm of chromosome 11 (11p15), encodes a C3HC4-type zinc finger protein motif (ring finger motif), and, consistent with a role in premeiotic or postmeiotic sperm development, one of its transcripts has been identified in abundance in the testicular tissue of fertile men, but neither in fetus nor in azoospermic patients. This suggested that *ZNF230* may be involved in spermatogenesis, and loss of its expression may lead to azoospermia (Zhang *et al.*, 2001). But so far, no clear biological function and mechanism had been elucidated.

In order to analyse the function of this novel zinc finger gene during vertebrate development, we decided to identify a potential orthologue in the teleost fish *Danio rerio*. This animal has become widely used as a genetic model to uncover specific functions of unknown proteins (Dooley and Zon, 2000; Rubinstein, 2003). Being transparent early embryonicl stages, easy to manipulate and highly reproductive makes the zebrafish an ideal animal system for molecular studies (Moro *et al.*, 2007).

We here report the cloning and characterization of an 816 bp cDNA sequence, named *rnf141,* which represents a candidate zebrafish orthologue of the human *ZNF230* gene. By using whole-mount *in situ* hybridization, RT-PCR, gene knockdown and overexpression analysis, we further showed its spatiotemporal expression pattern during early developmental stages.

## Materials and Methods

###  Zebrafish embryo maintenance

Zebrafish AB strain was provided by National Zebrafish Resources of China, and maintained under standard laboratory conditions at 28.5 °C (Westerfield, 1993). Embryonic stages were identified by morphological features (Kimmel *et al.*, 1995), and embryos in developmental stages of interest were fixed in 4% paraformaldehyde.

###  RNA extraction and reverse transcription

Total RNAs was isolated from adult zebrafish tissues using RNeasy Mini Kit (Qiagen) according to the manufacturer's instructions. SuperScript TM Reverse Transcriptase (Invitrogen) was used for reverse transcription.

###  Cloning of the zebrafish *rnf141* gene

Primers for 5'-RACE (366L 5'-ACGAGACGC CTCTACCATTCCATCC-3') and the other three pairs of primers (70U 5'-TCTCCATTTGGAGCCAAGATGGGC C-3' and 763L 5'- TAGATTTTTAAGGTCTGTGTGGG TG-3'; 338U 5'-AGGAGGATGGAATGGTAGAGGCGT C-3' and 619L 5'- GGCTCTGGCCGCTCCACTTGTCA AT-3'; and 432U 5'- TAGTTCAAATGTGGCGGCAG AGGGA-3' and 816L 5'- ATATATAGGTGGTCTTTT ATGGGGA-3') were designed to obtain the complete coding sequence, based on the potential orthologue of *ZNF230* in zebrafish. This orthologue sequence was acquired by PSI-BLAST alignment. 5'-RACE experiments were performed using SMART RACE cDNA Amplification Kit (Clontech) (Frohman *et al.*, 1988). cDNAs were reverse-transcribed from total RNAs of zebrafish tissue. The PCR products, including 5'- RACE products, were ligated into the pGEM-T Easy Vector (Promega), cloned and sequenced bidirectionally. Sequences obtained by 5'-RACE and the other three fragments were assembled, and the contig was queried to the zebrafish genome database to determine its chromosomal location, and analyse its genomic structure. The deduced amino acid sequence was searched against InterPro Database for possible functional domains.

###  Multi-tissue RT-PCR

To reveal the tissue distribution and expression of zebrafish *rnf141* gene, total RNA was extracted from embryos of various developmental stages (Kimmel *et al.*, 1995) and several tissues of adult zebrafish. The gene-specific primers 488U 5'-GGATGGGCAGGGTAAAAC AGTTGA- 3'(forward) and 683L 5'-GCATCCGACATG ACCCAGGATTCATTAG-3' (reverse) were designed to amplify a 196 bp fragment of *rnf141*. Amplification was performed in 30 cycles as follows: 30 s denaturation at 94 °C, 30 s primer annealing at 62 °C and 1 min extension at 72 °C. The PCR products were electrophoresed on 1% agarose gel in 1 x TAE buffer and ethidium bromide stained.

Primer sequences used for amplifying 470 bp ß-actin were 5'-TGTGGCCCTTGACTTTGAGCAG-3' (forward) and 5'-TAGAAGCATTTGCGGTGGACGA-3'(reverse), according to Kaslin *et al.*, (2004),. In negative controls, ddH_2_O was used instead of cDNA template. The gene-specific primers were selected from two exons separated by an intronic sequence to identify possible amplicons from contaminating genomic DNA. All synthetic oligonuleotides were purchased from Invitrogen Corporation (CA, USA).

### *rnf141* gene knockdown and overexpression experiments

*rnf141* morpholino antisense oligonucleotide (*rnf141*-MO, 5'-CCAGAAAGCTGCTGGCCCATCTTG G-3') was used to target *rnf141* mRNA, and 5'mispaired control morpholino (5'-CCAcAAAcCTcCTGcCCCATgT TGG-3') served as a control. Both were designed by using Gene-tools (Philomath, OR). The coding region of *rnf141* was ligated into vector pcDNA3 (Invitrogen) and linearized by appropriate restriction enzymes for mRNA was synthesis by using mMESSAGE mMACHINE® Kit (Ambion). *rnf141*-MO and control-MO were injected into 1-2 cell zebrafish embryos by using a Model PLI-90 Pico-Injector.

###  Whole-mount *in situ* hybridization

*rnf141* sense and antisense RNA probes were labeled with digoxigenin-11-UTP and synthesized by using DIG RNA Labeling Kit (SP6/T7) (Roche). The template for the probe was the entire 668 bp long cDNA. Whole-mount *in situ* hybridizations was performed as described by Westerfield (1995). Images were captured using an Olympus digital camera.

## Results and Discussion

###  Zebrafish *rnf141* has a C3HC4 zinc finger domain

We cloned zebrafish *rnf141* based on the amino acid sequence of human *ZNF230* and mouse *znf230* by PSI-BLAST alignment and RT-PCR including RACE. As a result, four fragments were obtained that formed a816 bp cDNA contig. This was submitted to GenBank (accession number: AY621088). Previous studies have shown that human *ZNF230* has two transcripts (Zhang *et al.*, 2001). In mouse, there are three transcripts of *znf230* (Qiu *et al.* 2003). In contrast, in our analyses on zebrafish we found only one transcript of this gene. Furthermore, our result was confirmed by another mRNA sequence record (GenBank accession numbe BC071534) that encodes the same protein submitted by Strausberg *et al.* Also, the sequence of our 3'-RACE fragment is identical to this sequence record.

**Figure 1:**
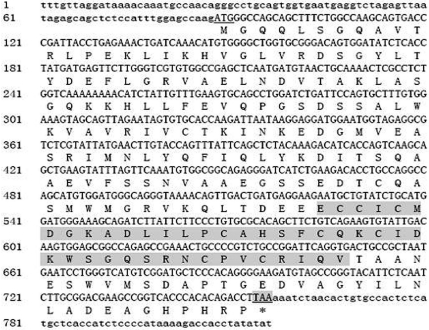
Nucleotide and predicted amino acid sequence of *rnf141* gene. The coding region (nucleotides 88-756) is in uppercase letters. The translation initiation codon is underlined. The stop codon at the 3'-end of the sequence is underlined and shaded. The deduced amino acid sequence (222 amino acids) is also shown below the nucleotide sequence. The predicted Ring-finger motif is shaded.

The predicted open reading frame from 88 to 756 is 668 bp in length and encodes a polypeptide of 222 amino acid residues with a C3HC4 zinc finger domain from 146 to 187 amino acid residues (Figure 1). Proteins with such a structure generally are nuclear transcriptional factors with the motifs being involved in both protein-DNA and protein-protein interactions. We found that zebrafish *rnf141* protein shares 75.65% and 75.22% identity in amino acid sequence with the human and mouse homologues, respectively. Furthermore, aligning amino acid sequence of zebrafish *rnf141* with *ZNF230* of other vertebrate species indicated that this gene domain is highly conserved (date not shown), suggesting functional similarity and conservation.

### *rnf141* is expressed in the CNS, primarily during early embryogenesis

**Figure 2:**
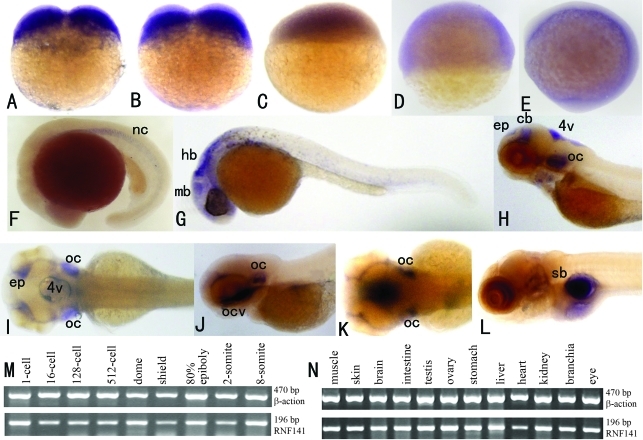
Expression analysis of *rnf141* in early embryos and adult zebrafish tissues. *rnf141* mRNA was initially detected at the 2-cell stage (A) and 4-cell stage (B), the signal become weaker at the sphere stage (C), shield stage (D) and bud stage (E). At the 5-somite stage (11.6 hpf), a characteristic pattern was displayed with marked staining in the notochord (F). At the 5-prim stage (24 hpf), a strong signal was detected in the head, particularly in the midbrain, hindbrain and the otic capsule (G). At the long-pec stage (48 hpf) the signal localization became restricted to the otic capsule, the 4^th^ ventricle, as well as the epiphysis and tegmentum (H-lateral view from left; I-dorsal view). An even more restricted expressions was detected in the oral cavity and otic capsule when embryos reached the mouth-protruding stage (72 hpf) (J- lateral view from left; K-dorsal view). An extensive expression in the gut and a restricted localization in swim bladder was found in 5 dpf embryo (L). Expression analysis of *rnf141* detected by RT-PCR in different developmental stage embryos (M) and adult zebrafish tissues (N). Abbreviations: mb, midbrain; hb, hindbrain; ep, epiphysis; cb, cerebellum; 4v, 4th ventricle; oc, otic capsule; ocv, oral cavity; sb, swim bladder.

**Figure 3:**
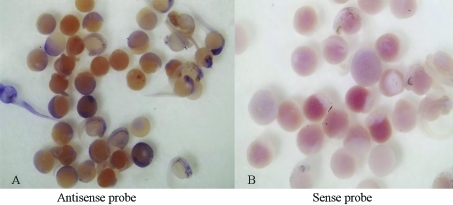
Whole-mount *in situ* hybridization with *rnf141* antisense and sense probe. Hybridizations were performed on two-cell stage to five-day-old embryos using *rnf141* antisense probe (A) and sense probe (B).

To analyse the spatiotemporal expression of *rnf141* during early embryonic development, whole-mount *in situ* hybridizations were performed on two-cell stage to five-day-old embryos using an antisense probe. As a result, *rnf141* transcripts were already detected at the two-cell stage (Figure 2A), thus suggesting a maternal origin of the transcript. From the sphere stage (4 hpf) to the tail bud stage (10 hpf) (Figure 2C-E), the *rnf141* transcripts have a broad distribution. However, at the 5-somite stage (11.6 hpf), a characteristic pattern was displayed with marked staining in the notochord (Figure 2F), and at the Prim-5 stage of the pharyngula period, this pattern was displayed in the midbrain and hindbrain (Figure 2G). Following the long-pec stage (48 hpf), restricted signal localization was evident in the otic capsule, 4th ventricle, epiphysis and cerebellum (Figure 2H-I). When embryos reached the protruding-mouth stage (72 hpf), obvious signals were detected in the oral cavity and otic capsule (Figure 2J-K). In 5 dpf (120 hpf) embryos, an extensive *rnf141* expression was visible in the gut, with a restricted localization in the swim bladder (Figure 2L). To assess the specificity of the antisense probe, a sense probe was used in a parallel control experiment at all stages. With this sense probe no staining was detected in any embryo (Figure 3).

The consistency of hybridization experiments was confirmed by RT-PCR expression analysis performed on cDNAs from whole zebrafish embryos at various early developmental stages (Figure 2M).

Since the 1 kb transcript of human *ZNF230* is only expressed in fertile male testis, whereas another 4.4 kb transcript was detected in many tissues; include heart, brain, skeletal muscle, kidney and pancreas (Zhang *et al.*, 2001), we further addressed the question as to whether zebrafish *rnf141* maintains its ubiquitous spatial expression in adult stages. As shown in Figure 2N, RT-PCR based analysis demonstrated that almost all analysed tissues of adult fish do display a high content of *rnf141* transcripts.

In conclusion, these results of whole-mount *in situ* hybridization and RT-PCR analyses performed both on zebrafish embryos and adult tissues provide evidence that *rnf141* may have multiple functions. The detection of its transcripts in the CNS of early embryos, especially restricted in the notochord at the 5-somite of the segmentation period, suggests a function for *rnf141* in zebrafish development. Further analysis is ongoing in order to improve knowledge on the role of *rnf141*.

### *rnf141* may play a part in normal dorsoventral patterning of zebrafish embryos

**Figure 4:**
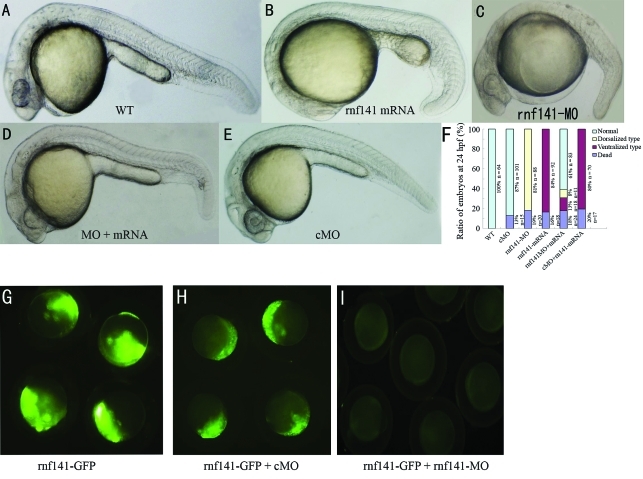
Knockdown and overexpression analysis of *rnf141*. All images are lateral views of live embryos at 24 hpf, anterior is to the left. (A) Wild-type embryo. (B) Injection with 200 pg *rnf141* mRNA resulted in enlargement of the yolk sac, in addition an extension and broadening of caudal ventral fin. (C) Injection with 12 ng *rnf141*-MO led to caudal ventral fin loss of and yolk sac extension. (D) Coinjection with 100 pg *rnf141* mRNA and 12 ng *rnf141*-MO resulted in a normal morphology. (E) Injection with 15 ng of a control morpholino, which differed from *rnf141*-MO in five mismatched nucleotides, did not show visible developmental defects. (F) The ratios of embryos showing different phenotype in experiments represented in A-E. Data were averaged from three independent experiments and expressed as means and standard deviations. The numbers of analysed embryos are indicated beneath each bar. (G-I) Live embryos at the shield stage, (G) embryos injected with 100 pg p*rnf141*-GFP DNA. (H) Embryos injected with 100 pg p*rnf141*-GFP DNA and 12 ng *rnf141*-5mis-MO. (I) embryos injected with 100 pg p*rnf141*-GFP DNA and 12 ng *rnf141*-MO.

**Figure 5:**
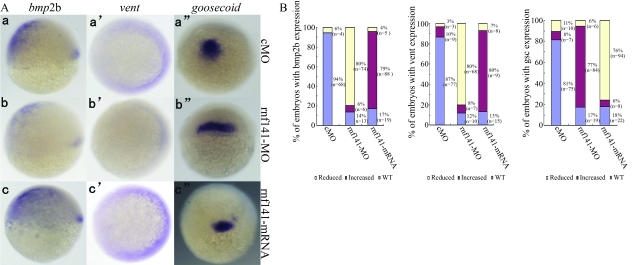
Expression patterns of marker genes in injected embryos at shield stage. (A) Expression patterns of *bmp2b* , *vent*, *goosecoid* in embryos injected with 15 ng *rnf141*-5mis-MO, 12 ng *rnf141*-MO, or 200 pg *rnf141* mRNA, respectively. For *bmp2b* expression, the embryo is shown in lateral view with dorsal pointed towards right; the embryo showing vent expression is depicted in animal pole view with dorsal oriented towards left; and the embryo with*goosecoid* expression is shown in a dorsal view with the animal pole pointed towards the top. (B) Statistical data for each marker gene of shown in A.

To further study the potential function of *rnf141*, we first injected zebrafish embryos with synthetic *rnf141* mRNA. Injection of 200 pg *rnf141* mRNA caused 84% (n = 92) of the embryos to show phenotypes that are characteristic of embryonic ventralization at 24 hpf (Figure 4B). The expression of the shield-specific gene *goosecoid* was decreased at the shield stage (Figure 5Ac”). In contrast, the ventral markers *bmp2b* and *vent* expanded dorsally during gastrulation (Figure 5Ac-c'). The ratios of embryos with altered marker gene expression are summarized in Figure 5B.

To investigate the role of endogenous *rnf141*, a morpholino antisense oligonucleotide (*rnf141*-MO) was injected into one-cell embryos. As a result, 81% (n = 88) of the embryos injected with 12 ng *rnf141*-MO exhibited dorsalized phenotypes at 24 hpf: complete loss of the yolk sac extension and partial loss of the caudal ventral fin (Figure 4C). The effects of *rnf141* knockdown on the expression of the marker genes *bmp2b*, *vent* and *goosecoid* (Figure 5Ab-b”) tended to be opposite to those of *rnf141* overexpression. In contrast, injection with 15 ng of control morpholino, which differs from *rnf141*-MO in five mismatched nucleotides, did not cause developmental defects (Figure 4E).

To test the efficiency of the morpholinos, fertilized eggs were injected with 12 ng *rnf141*-MO in combination with 100 pg of p*rnf141*-GFP DNA, an expression construct containing the full coding region of *rnf141* cDNA fused in-frame to a GFP coding sequence. At this dose of *rnf141*-MO, the injected embryos almost lacked green fluorescence from the GFP fusion protein (Figure 5I), while the same dose of *rnf141*-cMO injected embryos retained visible fluorescence (Figure 5H), suggesting that *rnf141*-MO could effectively block translation of *rnf141* mRNA.

To test the specificity of *rnf141*-MO, a 5 mis-pair *rnf141* mRNA corresponding to 5 mis-pair control morpholino was synthesized for rescuing the phenotype mediated with *rnf141*-MO. These results showed that the *rnf141*-MO-induced dorsalization could be neutralized by coinjection with a smaller amount of 5 mis-pair *rnf141* (Figure 4D), suggesting that *rnf141*-MO specifically targets *rnf141*.

In conclusion, knockdown of *rnf141* by using special morpholino-induced abnormal outcomes, including inordinate development of the CNS with an atrophic hindbrain, thin and crooked notochord, as well as disappearance of yolk sac extension, abnormality of axis, and partial loss of the caudal ventral fin. These embryos are characteristic of weakly dorsalized phenotypes, reminiscent of mini fin (*mfn*) and lost-a-fin (*laf*) mutant embryos, which were first described by Mullins *et al.* (1996) and were subsequently found to be caused by inefficient BMP signaling (Bauer *et al.*, 2001; Connors *et al.*, 1999; Mintzer *et al.*, 2001). Overexpressing this gene by injection of *rnf141* mRNA may caused embryos to show ventralized phenotypes. We also noted that *rnf141*-MO-induced dorsalization could be neutralized by coinjection of a smaller amount of *rnf141* mRNA, suggesting that *rnf141*-MO specifically targets *rnf141* mRNA. The expression of ventral markers (*bmp2b* and *vent*) and of a dorsal marker (*goosecoid*) were impacted by altered expression of *rnf141*, thus suggesting that zebrafish *rnf141* may participate in normal dorsoventral embryonic patterning. Further research is needed to better understand the respective biological pathway(s) and improve the knowledge on the function of *rnf141*.
